# Internal friction controls active ciliary oscillations near the instability threshold

**DOI:** 10.1126/sciadv.abb0503

**Published:** 2020-08-12

**Authors:** Debasmita Mondal, Ronojoy Adhikari, Prerna Sharma

**Affiliations:** 1 Department of Physics, Indian Institute of Science, Bangalore, Karnataka 560012, India; 2 The Institute of Mathematical Sciences–Homi Bhabha National Institute, Chennai 600113, India.; 3 Department of Applied Mathematics and Theoretical Physics, Centre for Mathematical Sciences, University of Cambridge, Cambridge CB3 0WA, UK.

## Abstract

Ciliary oscillations driven by molecular motors cause fluid motion at micron scale. Stable oscillations require a substantial source of dissipation to balance the energy input of motors. Conventionally, it stems from external fluid. We show, in contrast, that external fluid friction is negligible compared to internal elastic stress through a simultaneous measurement of motion and flow field of an isolated and active *Chlamydomonas* cilium beating near the instability threshold. Consequently, internal friction emerges as the sole source of dissipation for ciliary oscillations. We combine these experimental insights with theoretical modeling of active filaments to show that an instability to oscillations takes place when active stresses are strain softening and shear thinning. Together, our results reveal a counterintuitive mechanism of ciliary beating and provide a general experimental and theoretical methodology to analyze other active filaments, both biological and synthetic ones.

## Teaser

Internal friction in cilia dominates because fluid friction is small compared to elastic stresses caused by active motors.

## INTRODUCTION

Cilia and flagella are prototypical engines of micron-scale motility, used by the biological world in myriad contexts (*1*, *2*). They are classic examples of nonequilibrium active filaments that undergo spontaneous oscillations by converting stored or ambient energy into mechanical motion (*3*). Their elemental structure, called axoneme, consists of cross-linked microtubule (MT) doublets and dynein motors, which apply forces on MT, through adenosine triphosphate (ATP) hydrolysis, to cause periodic bending of the whole structure (*4*). In addition to its importance in the cellular context, ciliary beating has been mimicked in synthetic filaments for applications in cargo transport, microfluidics, and drug delivery (*5*, *6*).

Naturally, there have been a number of studies devoted to understand the microscopic mechanisms of ciliary oscillations (*2*, *7*–*12*). Most of them analyze beating based on passive elastic stresses calculated from measured filament waveform, models of active drive, and dissipation stemming from external fluid. The dissipation is usually computed using slender body integral equations since filament waveform alone cannot be used to validate its form. Intuitively, hydrodynamics should play an essential role as cilia operate in the regime of low Reynolds number (∼10^−3^). However, its contribution to synchronization and collective behavior of cilia is highly debated because most of these in vivo experiments are conducted on live cells where it is difficult to delineate hydrodynamics from other coupling mechanisms such as cell body rocking or intracellular means (*13*–*17*). In addition, biopolymers often have substantial internal friction (*18*). Hence, to determine the dominance or lack of hydrodynamics in ciliary oscillations, an accurate measurement of the external viscous drag and its competition with the other elastic stresses for an in vitro system of cell-free isolated cilium is required, where cell body rocking and intracellular basal body coupling are eliminated. This can be accomplished by measuring its flow field along with the waveform.

Here, we present the first simultaneous measurement of the bending waveform and flow field of an isolated and active *Chlamydomonas* axoneme, beating near the critical ATP concentration at which oscillations set in, at high spatiotemporal resolution to address the role of external fluid in its beating. Our measurements demonstrate that hydrodynamic dissipation, accurately described by resistive force theory (RFT), is negligible compared to internal elastic stresses. Consequently, a dissipation mechanism internal to the filament is essential for stable driven oscillations, in contrast to the widely held view that fluid friction is the only important source of dissipation (*9*, *12*). We combine these insights with a theoretical model of filament motion that includes a minimal spring-dashpot form of active stress. We show that there exist critical values of active stress beyond which the model exhibits oscillations, namely, active stresses should be strain softening and shear thinning.

## RESULTS

### Experimental system

**Fig. 1:**
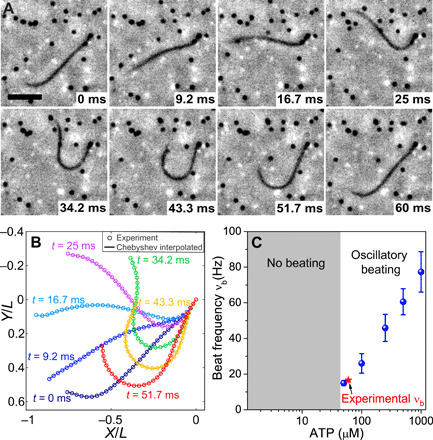
Simultaneous measurement of motion and flow. (**A**) Time lapse snapshots of a clamped and reactivated *Chlamydomonas* axoneme at 60 μM ATP in the presence of tracer particles (black dots). Scale bar, 4.2 μm. (**B**) Nondimensionalized experimental positions and their corresponding Chebyshev interpolants. (**C**) Beat frequency of axonemes as a function of ATP concentration (blue circles). Red star indicates experimental conditions that are near the threshold for the onset of oscillations.

Isolated and reactivated axonemes of ~11 μm length and ~0.2 μm diameter (*4*), purified from the unicellular algae *Chlamydomonas reinhardtii*, are clamped at one end on a glass coverslip. Their oscillations, with beat frequency ν*_b_* ∼ 16.22 Hz, are approximately planar with an average centerline height *h* ∼ 0.9 μm from the surface. Passive microspheres are introduced into the suspension as tracers for measuring flow of the ambient fluid using particle tracking velocimetry (PTV) (see Materials and Methods). We capture motion of both the axoneme and tracers at high spatiotemporal resolution using a 60× phase objective coupled with a high-speed camera (Fig. 1A and movie S1). The detailed experimental procedure is described in Materials and Methods. The position of the axoneme is sampled at many points over its length to construct a global Chebyshev polynomial interpolant of the parametric form ***R***(*s*) = S ***a****_n_*(*t*)*T_n_*(*s*), where 0 ≤ *s* ≤ *L* is the arc length and ***a****_n_* is a vector of Chebyshev coefficients for the *x* and *y* components of the position (Fig. 1B).

### Filament geometry and mechanics

We perform experiments near the critical ATP concentration at which the axoneme transitions from quiescent to oscillatory state through an instability threshold (Fig. 1C) (*19*). Near this threshold, details of the internal structure of the axoneme become irrelevant. Therefore, we model it as an inextensible, but shearable, active rod of uniform circular cross section of diameter *a* and length *L*, with a centerline described by the curve ***R***(*s*) to calculate stresses averaged over its cross section. We attach an orthogonal Frenet-Serret frame to each point of the curve. Planar motion of the filament results in the curve tangent to be parameterized by the tangent angle θ as ***t***(*s*) = [cos θ(*s*), sin θ(*s*)], which automatically satisfies the inextensibility constraint, ***t*** · ***t*** = 1. The position of the curve is obtained in terms of the tangent angle by integrating ∂_s_***R*** = ***t***. The shear strain of the rod is *u*(*s*), which is assumed to vanish at the clamped base, *u*(0) = 0. Inextensibility then requires the shear strain to accommodate the filament bending as *u*(*s*) = *a*[θ(*s*) − θ(0)] ≡ *a*Δθ(*s*), and the kinematics of the filament is completely specified by θ or equivalently by shear angle, Δθ (*7*). Notably, the unobservable shearing of the filament can be inferred from its observable curvature, κ = *∂_s_*θ ≡ *∂_s_*Δθ. There is no assumption of small curvature in this kinematic description. Internal active stresses cause the filament to shear and, by the kinematic constraint, to curve.

We assume that the rod supports an internal stress whose stress and moment resultants on the cross section at *s* are, respectively, ***F***(*s*) and ***M***(*s*) and that it is acted upon by forces and moments whose sum per unit length are, respectively, ***f*** and ***m***. Then, in the absence of inertia, the balance equations for force and torque are (*20*)

$$\partial_{s}F+f=0,\;\;\partial_{s}M+t\times F+m=0$$ (1)

Internal moments included in ***m***, which can exist only if the internal stress is antisymmetric, are generally omitted in the elasticity of rods but are essential to our model (*20*). The above equations are closed by identifying the relevant forces, moments, and their constitutive equations in terms of the kinematic variables. Integrating the force equation, the stress resultant can be expressed as $F(s)=\int_{s}^{L}f(s)\mathrm{ds}+F(L)$, where ***F***(*L*) = 0 at the free terminus of the rod. The only force per unit length relevant here is the external hydrodynamic drag, ***f****^v^*.

### External viscous drag

A rod moving through a viscous fluid experiences a drag ***f****^v^*(*s*) and creates a flow ***v***(***r***). In the experimentally relevant limit of slow viscous flow and a slender rod, *L* ≫ *a*, the integral representation of Stokes equation gives

$$v(r)=-\int_{0}^{L}G(r,R(s))\cdot f^{v}(s)\;\mathrm{ds}$$ (2)

This represents a distribution of Stokeslets of strength ***f****^v^*(*s*), where ***G*** is a Green’s function of the Stokes equation. The matching of the fluid flow to the velocity of the rod at its surface yields the slender body integral equation whose formal solution is $f^{v}(s)=-\int_{0}^{L}\gamma (s,s^{\prime })\cdot \overset{\cdot }{R}(s^{\prime })\;ds^{\prime }$. The friction kernel is often approximated by a local form with a constant friction coefficient, **γ**(*s*, *s*′) = **γ**δ(*s* − *s*′). In this RFT limit, the drag is

$$f^{v}(s)=-\gamma \cdot \overset{\cdot }{R}(s),\gamma =\gamma_{n}n⊗n+\gamma_{t}t⊗t$$ (3)

where γ*_n_* and γ*_t_* are the normal and tangential components of the friction coefficient, respectively, and $\overset{\cdot }{R}={\overset{\cdot }{R}}_{n}n+{\overset{\cdot }{R}}_{t}t$ is the centerline velocity of the rod in terms of its normal and tangential components.

**Fig. 2:**
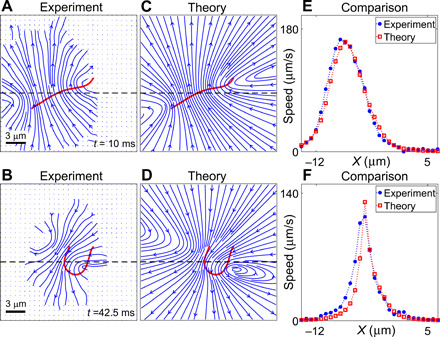
External drag force. Experimentally measured instantaneous flow fields using PTV during (**A**) power and (**B**) recovery stroke for the corresponding initial axonemal conformations (red lines). Streamlines represent flows with *v* > 8 μm/s and vector fields for lower speeds in the Brownian noise regime. (**C** and **D**) Theoretically computed flow fields using RFT corresponding to (A) and (B), respectively. (**E** and **F**) Comparison of experimental and theoretical flow fields along representative cuts (dashed lines).

With the viscous drag thus determined in terms of the centerline velocity, we now validate RFT using experimentally measured instantaneous flow fields (Fig. 2, A and B). We compare these experimental flows with theoretically computed ones using Eqs. 2 and 3 (Fig. 2, C and D), where $\overset{\cdot }{R}$ is determined from the measured waveform, γ*_n_* = 4πη/ *ln* (4*h*/*a*) = 4.35 mPa·s, γ*_t_* = γ*_n_*/2 (*21*) (fluid viscosity, η = 1 mPa.s), and ***G*** is the Lorentz-Blake tensor for flow near a no-slip wall (*22*). Representative cuts along the experimental and theoretical flows show that there is a good agreement between the two (Fig. 2, E and F). A more comprehensive comparison is given by root mean square deviation of the flows, $\text{RMSD}=\sqrt{\sum_{i=1}^{\mathrm{NS}}{\left(v_{i}^{\text{expt}}-v_{i}^{\text{th}}\right)}^{2}/\text{NS}}$, where NS is the number of grid points and $v_{i}^{\text{expt}}$ and $v_{i}^{\text{th}}$ are the experimental and theoretical flow magnitudes at the *i*th grid point, respectively. RMSD of *v_x_*, *v_y_*, and ∣***v***∣ in Fig. 2 are 4.32, 8.28, and 7.47 μm/s (A and C) and 7.26, 7.89, and 9.81 μm/s (B and D), respectively, all of which are within the Brownian noise regime implying a good match. Therefore, we have now verified by direct measurement that the hydrodynamic drag force is unambiguously given by RFT. This form of drag is used to evaluate the stress resultant, ***F***, and its normal component, *F_n_*, is used in the torque balance equation below.

### Scalar equation of motion of the filament

In slow viscous flow as is shown above, the drag acts in the plane of motion, and the stress resultant remains in that plane. Therefore, the couple resultant ***M*** is normal to the plane of motion, along the frame binormal ***b***, and vanishes at the free terminus, giving *M*(*L*) = 0. As the only nonzero components of ***t*** × ***F*** = *F_n_****b*** and ***m*** are normal to the plane, torque balance reduces to a scalar equation, *∂_s_M* + *F_n_* + *m* = 0. The dissipation in this equation is contained in the normal component of the stress resultant due to external viscous drag as $F_{n}(s)=n(s)\cdot \int_{s}^{L}f^{v}(s\prime )\mathrm{ds}\prime =-\gamma_{n}g_{n}^{\prime }(s)$ where $g_{n}^{\prime }(s)=n(s)\cdot \int_{s}^{L}[{\overset{\cdot }{R}}_{n}(s\prime )n(s\prime )+{\overset{\cdot }{R}}_{t}(s\prime )t(s\prime )/2]\mathrm{ds}\prime$ using Eq. 3. Here, the filament velocity components can be expressed in terms of the tangent angle, θ, using R·(s)=∫0st·(s')ds'. The simplest elastic contributions for an inextensible and shearable active rod are that due to bending, *M* = *EI*κ, and shear, *m* = −*aku* (*7*). The source of filament motion is included as the active moment per unit length, *m*^A^. Material parameters in these constitutive relations are bending rigidity, *EI* = 600 pNμm^2^ (*12*, *23*), and shear stiffness, *k* = 2000 pN/μm^2^ (*23*, *24*). Torque balance, closed by the constitutive relations and the expression for *F_n_*, yields the following dynamical equation

$$\mathrm{EI}\partial_{s}\kappa -\mathrm{aku}-\gamma_{n}g_{n}^{\prime }+m^{A}=0$$ (4)

**Fig. 3:**
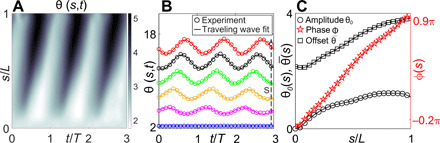
Traveling wave parameterization to tangent angle. (**A**) Space-time plot of the experimental θ. (**B**) Traveling wave fit to experimental θ at representative arc lengths, *s*. Plot for θ at each *s* is shifted along the *y* axis, for clarity. (**C**) Amplitude, phase, and offset of the traveling wave in θ along *s* and their corresponding Chebyshev interpolants (solid lines).

All the passive terms in this equation can be expressed completely in terms of the tangent angle, θ. Experimental data reveal that the tangent angle can be parameterized by a traveling waveform, $\theta (s,t)=\theta_{0}(s)\text{sin}\;[\omega t-\phi (s)]+\bar{\theta }(s)$, where θ_0_, ω, ϕ, and $\bar{\theta }$ are the amplitude, angular frequency, phase, and offset, respectively (Fig. 3) (*8*, *12*). As we are interested in oscillations about the time-averaged shape of the beat, $\bar{\theta }$, we define $\theta^{\prime }(s,t)=\theta (s,t)-\bar{\theta }(s)$. In the following, we focus on θ^′^ and drop the prime such that θ(*s*, *t*) ≡ θ_0_(*s*) sin [ω*t* − ϕ(*s*)], which represents the dynamic oscillatory beat of the filament about the mean shape. Therefore, we use this parametric form, instead of the Chebyshev interpolant, to estimate all angle-dependent quantities in the dynamical equation.

The dimensionless equation of motion with $l_{\kappa }=\sqrt{\mathrm{EI}/a^{2}k}=2.74$ μm as the curvature penetration length scale and $\nu_{h}=\mathrm{EI}/\gamma_{n}l_{\kappa }^{4}=2447$ Hz as the hydrodynamic relaxation frequency scale of the system is

$$\partial_{s}^{2}\Delta \theta -\Delta \theta -g_{n}+m^{A}=0$$ (5)

**Fig. 4:**
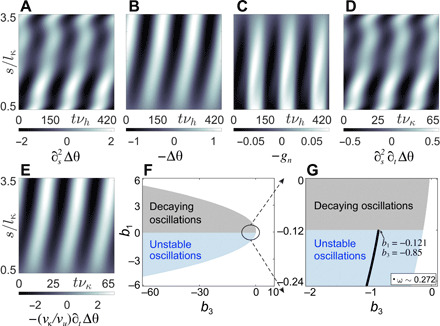
Dynamics and linear stability analysis. Space-time plots of passive moments per unit length contributing to (**A**) bending elasticity, (**B**) shear elasticity, and (**C**) normal component of the stress resultant, which includes external viscous drag; (**D**) bending friction and (**E**) shear friction. Scaling parameter, $\mathrm{EI}/l_{\kappa }^{2}=80\text{pN}$. (**F**) Oscillatory nature of the complex frequency, *z*, from linear stability analysis of Eq. 7 in the parameter space (*b*_3_, *b*_1_) for the fundamental mode, *q*_1_. No oscillatory solutions exist in the white patches. (**G**) Zoomed-in view of (F) near the instability threshold, where ω denotes the oscillation frequency.

Here, $g_{n}=g_{n}^{\prime }/l_{\kappa }^{2}\nu_{h}$ and *m*^A^ is rescaled by $l_{\kappa }^{2}/\mathrm{EI}$. Using θ from the traveling wave parameterization of experimental data and constitutive parameters from the literature, the space-time variation of elastic and viscous terms of Eq. 5 is plotted in Fig. 4 (A to C). It shows that the nonlinear viscous dissipation has a standing wave component in contrast to the linear elastic terms (*8*). On comparing their colorbars, we conclude that the hydrodynamic dissipation, *g_n_* ∼ O(0.01), is negligible compared to the elastic forces, which are of O (*1*).

The axoneme consumes energy in the form of ATP and exhibits stable oscillations. Such a continuously driven system can be in a dynamical steady state only when the elastic stresses due to the drive are balanced by some dissipation. Since external fluid friction cannot account for this dissipation, consistency demands that the internal stress has, in addition to an elastic component, a dissipative frictional component. Each kinematic degree of freedom, i.e., bending and sliding, can contribute to dissipation. Notably, bending friction in MTs has been experimentally demonstrated to be dominant over hydrodyamic friction for length scales smaller than 20 μm and attributed to slow structural rearrangements (*18*, *25*, *26*). The bending friction coefficient of an 11 μm-long axoneme, Γ_κ_ = 1.6 pNμm^2^s, is the same as that of a single MT since it is an intensive quantity (*25*, *26*). Although there is no experimental measure of the shear friction coefficient of an axoneme, Γ*_u_*, several experimental studies suggest the presence of inter-MT sliding friction (*24*). We consider Γ*_u_* = 10 pNs/μm from estimates of nexin protein friction (see table S1) (*27*). Earlier work introduced similar terms for internal viscous stresses for either stabilization of the numerical simulations of bend propagation in active filaments (*9*, *28*–*30*) or on the basis of theory alone (*11*, *31*). However, here, we have shown experimentally that such terms are necessary to completely account for dissipation in ciliary motion.

### Dynamical equation without external friction

We, therefore, neglect the external viscous drag and include the internal viscous stresses to rewrite the scalar torque balance equation, *∂_s_M* + *m* = 0. Hence, the constitutive equations are modified as $M=\mathrm{EI}\kappa +\Gamma_{\kappa }\overset{\cdot }{\kappa }$ and $m=-\mathrm{aku}-\Gamma_{u}\overset{\cdot }{u}+m^{A}$. The modified dynamical equation in terms of the shear angle, Δθ, is $\mathrm{EI}\partial_{s}^{2}\Delta \theta +\Gamma_{\kappa }\partial_{s}^{2}\partial_{t}\Delta \theta -a^{2}k\Delta \theta -a\Gamma_{u}\partial_{t}\Delta \theta +m^{A}=0$. The negligible role of fluid friction leads to a second-order reaction-diffusion equation for the shear angle rather than fourth-order partial differential equations that are commonly obtained when fluid friction is retained (*10*, *11*, *29*). Identifying the two frequency scales of the system, a curvature relaxation frequency scale, ν_κ_ = *EI*/Γ_κ_, and a sliding relaxation frequency scale, ν*_u_* = *ak*/Γ*_u_*, we note that ν_κ_/ν*_u_* = 9.38, i.e., both kinematical degrees of freedom contribute to dissipation. The dimensionless equation of motion with ν_κ_ as the frequency scale is

$$\partial_{s}^{2}\Delta \theta +\partial_{s}^{2}\partial_{t}\Delta \theta -\Delta \theta -\frac{\nu_{\kappa }}{\nu_{u}}\partial_{t}\Delta \theta +m^{A}=0$$ (6)

where *m*^A^ is rescaled by $l_{\kappa }^{2}/\mathrm{EI}$. Figure 4 (D and E) shows the variation of the internal viscous stresses over three beat cycles along the filament length. The colorbars in Fig. 4 (A to E) indicate that the internal viscous stresses due to bending friction of O(0.1) and shear friction of O(1) compete with the elastic stresses of O(1), unlike the negligibly small external drag of O(0.01).

The internal passive stresses being completely defined, a constitutive relation for the active moment in terms of kinematic variables is required. The axoneme having both elastic and viscous material parameters, the motor activity will induce a viscoelastic response. Hence, we assume a minimal spring-dashpot form for the dynamics of *m*^A^, *∂_t_m*^A^ + *b*_1_*m*^A^ = *b*_3_Δθ (*19*, *32*), whose Fourier representation describes the spring-like elastic and dashpot-like viscous dampening response of the system under oscillating shear as shown later. Here, the active stress is parameterized by two constants: *b*_1_ controls the autonomous dynamics of *m*^A^, and *b*_3_ controls the amount of feedback it receives from the sliding kinematics. We have chosen this simplest form of constitutive relation as it is first order in time, lowest order in wave number, and linear in Δθ. This minimal constitutive relation is most relevant for a coarse-grained description of axoneme as used here. This relation for *m*^A^ together with Eq. 6 forms a pair of coupled equations

$$\partial_{t}[\begin{array}{c} (\partial_{s}^{2}-\nu_{\kappa }/\nu_{u})\Delta \theta \\ m^{A} \end{array}]=[\begin{array}{cc} 1-\partial_{s}^{2} & -1\\ b_{3} & -b_{1} \end{array}][\begin{array}{c} \Delta \theta \\ m^{A} \end{array}]$$ (7)

which emphasizes that the passive and active parts in this model are independent, but coupled, degrees of freedom.

These dissipative linear partial differential equations can sustain stable oscillations only in the presence of nonlinearities whose choice will become crucial far from the instability threshold (*11*, *19*, *33*). We now focus on the linear regime and seek to identify the threshold for the onset of oscillations and its frequency near threshold. This is relevant to our experimental results because the axoneme in this study beats at 60 μM ATP, near the critical ATP concentration of 45 μM at which oscillations set in (Fig. 1C). Hence, the axoneme is beating near the instability threshold, where the nonlinearity is weak and the oscillation frequency of the limit cycle is that of the linear analysis evaluated at the threshold (*19*, *33*).

We perform linear stability analysis on the coupled Eq. 7 in the Fourier domain (see section S2 for detailed analysis). The dispersion relation is quadratic in complex frequency *z*, $[(1+q_{n}^{2})-\mathrm{iz}(q_{n}^{2}+\nu_{\kappa }/\nu_{u})](\mathrm{iz}-b_{1})+b_{3}=0$. The clamped head-free end boundary conditions of the filament discretize the wave numbers to $q_{n}=\frac{n\pi }{2(L/l_{\kappa })}$ for odd *n*. Unstable oscillations are possible only when both *b*_1_ < 0 and *b*_3_ < 0 (Fig. 4F). These parameters can alternatively be related to the elastic, *G*′, and viscous, *G*′′, response coefficients of the active stress through its fundamental Fourier mode of oscillation frequency ω as $\tilde{m^{A}}=(G\prime +i\omega G^{\prime \prime })\tilde{\Delta \theta }$, where $G\prime =b_{1}b_{3}/(b_{1}^{2}+\omega^{2})$ and $G^{\prime \prime }=-b_{3}/(b_{1}^{2}+\omega^{2})$ (*10*, *19*). The sign of response coefficients determines the nature of active stress. If *G*′, *G*′′ < 0, the system’s passive spring constant and friction coefficient get renormalized by the ATP-dependent dynein activity. In our case, *b*_1_, *b*_3_ < 0 ⇒ *G*′, *G*′′ > 0, i.e., activity works against the material response and leads to strain softening (*G*′ > 0) and shear thinning (*G*′′ > 0) in the system (see section S3 for detailed discussion). The experimental beat frequency of the axoneme (ω_expt_ = 2πν*_b_*/ν_κ_ = 0.272), operating close to the threshold, constrains the parameters of the constitutive relation at hand, namely, *b*_1_ = − 0.121, *b*_3_ = − 0.85 for the fundamental oscillatory mode (Fig. 4G). Elastoviscous response coefficients of the active stress computed with these values, *G*′ = 1.16, *G*′′ = 9.6, imply that the viscous response of the active stress dominates the elastic one in our experiment. On comparing the nature of active stress so obtained with a microscopic model of load-dependent detachment of motors in the experimentally relevant dominantly linear regime of the post-threshold dynamics (*10*, *19*, *33*), we infer that axonemal dyneins are low-duty ratio motors (see section S4 for details), which agrees with previous studies (*21*).

We emphasize again that the existence and dominance of internal friction over hydrodynamic drag in isolated ciliary dynamics is borne out of experimental measurements alone and not through detailed modeling. Simultaneous measurement of flow field and waveform of an active cilium gives us the unambiguous measure of the hydrodynamic drag force, i.e., the external fluid friction. The passive internal elastic stresses are calculated from the experimental waveform using minimal constitutive relations for bending and shear elasticity, which are widely accepted in the literature for elasticity of rods. Comparing the experimentally measured fluid friction with that of the passive elasticity led to the conclusion that fluid friction is not enough to counteract the elastic stresses due to the active drive, and consequently, stable ciliary oscillations need internal friction to reach their dynamical steady state. The above theoretical analysis of the filament equation of motion using a minimal constitutive relation for the active drive is simply to illustrate that oscillations exist in the presence of internal friction, too.

## DISCUSSION

In conclusion, to the best of our knowledge, this is the first direct experimental evidence of the negligible role of external fluid friction in ciliary oscillations near the instability threshold, thereby suggesting that internal dissipation mechanisms are essential to have a self-consistent understanding of ciliary beating. Our results are complementary to studies conducted on live cells/sperms (*34*) and reactivated cilia at high ATP conditions (*35*) wherein changing the fluid viscosity alters both waveform and beat frequency of cilia by about 20%. At low ATP concentration investigated in this study, ciliary beat frequency and waveform are almost independent of the viscosity of ambient fluid (*35*). It is crucial to study this regime near the instability threshold because, here, the system is governed by linear terms that are generic and do not depend on the minor structural details of the system, leading to an understanding that is universal in nature (*19*). As in this study, our results imply that one should include the contribution of internal friction with the obvious source of external fluid friction in constructing balance equations for active filaments in viscous fluids to correctly understand the dynamical regimes in which they operate.

This result will inspire further studies on the role of fluid friction in ciliary beating far from the threshold and in collective behavior of cilia. In addition, the measured flow field can distinguish internally active filaments from those driven by surface forces, for example, in phoretic chains (see fig. S1) (*36*). Therefore, a measurement of the flow field of an oscillating cilium provides vital information on its mechanisms of operation that cannot be obtained from the measurement of motion alone. Our forward approach of using the experimental insights from simultaneous waveform and flow measurements to build a theoretical model of active filament is in contrast to the existing reverse approaches of starting with microscopic models of active stress to match the observed macroscopic waveform of the filament (*10*–*12*). Lastly, our approach can serve as a paradigm for analysis and regulation of any active slender body, both biological and synthetic one, in a viscous fluid.

## MATERIALS AND METHODS

### Axoneme purification and reactivation

Wild-type *C. reinhardtii* cells (strain: CC1690) are synchronously grown in 12:12 hours light:dark cycle in TAP+P medium (tris acetate phosphate) (*37*). They are collected 2 to 3 hours after the beginning of light cycle, at OD_750_ (optical density at 750 nm) ≈0.15 to 0.25, followed by washing in HES buffer [10 mM Hepes (pH 7.4), 1 mM EGTA, and 4% sucrose] thrice. The plasma membrane covering the cells is disrupted by adding 0.15% of a nonionic detergent IGEPAL CA-630 (I8896, Sigma-Aldrich) in HMDEK buffer [30 mM Hepes (pH 7.4), 5 mM MgSO_4_, 1 mM dithiothreitol (DTT), 1 mM EGTA, and 50 mM K-acetate] to the cell pellet. The cells in IGEPAL are then kept in ice for ∼5 to 7 hours. These cells are devoid of cell membranes and are called cell models. Some of them shed their demembranated flagella, called axonemes, due to weakening of the attachment to the cell body. Isolated axonemes are then separated from the cell models by centrifugation. Axonemes are then mixed with 30% saturated sucrose, flash-frozen, and stored at −80^∘^C for long-term usage.

This method of purification, by long-term exposure to detergent, yields nonsticky axonemes, in contrast to the commonly used dibucaine procedure (*37*, *38*), hence, essential for flow field measurements. These nonmotile axonemes regain their motility in the presence of 45 μM to 1 mM ATP in HMDEKP buffer (HMDEK + 1% PEG-20k) and are said to be reactivated. There was no distinction in the nature of beating of the frozen axonemes, when thawed, from those that were not frozen. We use an ATP regeneration system, to hold the ATP concentration constant within the sample for approximately 40 min, enough for imaging approximately six to seven isolated axonemes in one sample. The ATP regeneration system composes of 6 mM sodium creatine phosphate (27920, Sigma-Aldrich) and 40 U/ml creatine phosphokinase (CPK) from bovine heart (C7886, Sigma-Aldrich). The commercially available CPK is typically oxidized; hence, we reduce it by DTT at room temperature for efficient reactivation (*39*).

### Tracer particles

The tracer particles chosen, for flow field measurements, are neutrally buoyant (polystyrene microspheres with a density of 1.055 g/cc) and small (diameter of 200 nm, the lowest size that can be used with diffraction-limited optical imaging) so that their motion is nearly identical to the fluid in which they reside. Commercially available charge-stabilized microspheres stick to each other and to the axoneme due to screening of electrostatic interactions by the divalent ions and salts present in the reactivation buffer. We graft long chains of block copolymer PLL-PEG20k [poly-l-lysine (PLL), P7890, 15 to 30 kDa, Sigma-Aldrich; mPEG-SVA-20k, NANOCS] onto 200-nm negatively charged sulfate latex beads (S37491, Thermo Fisher Scientific) to impart additional steric stabilization.

### Surface modification of glass surfaces and sample preparation

The coverslips and slides are cleaned with a hot soap solution (1% Hellmanex III), followed by rinsing with ethanol and 100 mM potassium hydroxide. We graft polyacrylamide brush on these clean glass surfaces to suppress depletion interaction of beads and filament with the surfaces. The coverslips are further modified with randomly located sticky patches of 0.05% PLL over the polyacrylamide brush, to clamp the axonemes at one end. We introduce the reactivation buffer containing axonemes and tracer particles inside a sample chamber made up of a glass slide and coverslip sandwich with double-sided tape with a thickness of 65 μm as the spacer.

### Imaging and depth of focus

The sample is mounted on an inverted microscope (Olympus IX83), equipped with 60× oil immersion phase objective [0.65 to 1.25 numerical aperture (NA), UPlanFL N, PH3] and connected to a high-speed complementary metal oxide semiconductor (CMOS) camera (frame rate, 1200 frames per second; Phantom Miro C110, Vision Research) for imaging. We use an intermediate NA between 0.65 and 1.25 for the 60× variable NA phase objective, to capture most of the filament beat in focus. We have measured the depth of focus (Δ*Z*) of the objective at this intermediate NA to be 1.4 μm. We only image time lapse sequences of those axonemes that are clean, clamped at one end with proper beating, having 80 to 90% of the filament in focus at a Δ*Z* of 1.4 μm, and do not have another reactivated filament in the surrounding area of 30 μm × 30 μm. The frame rate in the high-speed imaging is suitably chosen to capture approximately 1000 beat cycles per axoneme, with ∼55 to 75 conformations per beat cycle, for example, 1200 frames per second for the axoneme in movie S1, whose beat frequency is approximately 16.63 Hz. Furthermore, the theoretically computed flow field is also depth averaged over this Δ*Z* for appropriate comparison with the experimental flow field.

### Extracting filament conformation from images

We manually identify position coordinates of the axoneme for three beat cycles from the recorded time lapse sequences, which are then smoothened along *s* and *t* by using Savitzky-Golay filter of orders 3 and 5, respectively. The filament positions ***R***(*s*) and arc length *s* are nondimensionalized with the filament length *L* and time *t* with beat period *T*, followed by setting the clamped end of the filament to (0,0). These nondimensionalized experimental positions are used to construct their Chebyshev interpolant as mentioned in the Results section. Higher-order derivatives of the interpolation lose accuracy at the ends of the axoneme and data from those parts are, accordingly, discarded (fig. S2). Hence, we consider *s*/*L* ∈ [0.1,0.9] when computing derivatives higher than first order and neglect the end values (as shown in Fig. 4, A to E).

### Particle tracking velocimetry

The recorded movies are background subtracted, before tracking the tracer displacements, to consider only those tracers that are not stuck to the coverslip and are following the flow. We track the tracer displacements in between two filament conformations with an infinitesimal time gap, Δ*t* (for example, Δ*t* ≈ 3.33 ms for power stroke waveforms and Δ*t* ≈ 4.98 ms in revovery stroke waveforms). As the filament has a nonuniform beat frequency *ν_b_* = 16.63 ± 0.62 Hz, more than ∼940 beat cycles, each conformation will not exactly repeat itself after one beat period. Hence, we cross-correlate each filament conformation for a given beat period with the whole recorded sequence of ∼940 beat cycles. The correlation peaks indicate the frames that have similar conformation. The stack of matched conformations is checked manually to delete any conformation with more than 10% dissimilarity. The displacement of tracers in between these two conformations is calculated using standard MATLAB tracking routines, and velocity vectors are obtained from ∼500 to 700 beat cycles. The resulting velocity vectors are placed on 29 × 29 mesh grid, each of size 0.74 μm × 0.74 μm over the image, and the mean at each grid point is computed. The gridded velocity vectors are further smoothened using a 2 × 2 point averaging filter.

## Supplementary Material

abb0503_SM.pdf

abb0503_Movie_S1.avi

## SUPPLEMENTARY MATERIALS

Supplementary material for this article is available at http://advances.sciencemag.org/cgi/content/full/6/33/eabb0503/DC1
