# Supplementary material for: Internal friction controls active ciliary oscillations near the instability threshold
Source: Sci Adv. 2020 Aug 12;6(33):eabb0503. doi: 10.1126/sciadv.abb0503 (PMC7423358; doi:10.1126/sciadv.abb0503)
Supplement: abb0503_SM.pdf [file abb0503_SM.pdf]

Science Advances
AAAS

advances.sciencemag.org/cgi/content/full/6/33/eabb0503/DC1

Supplementary Materials for

Internal friction controls active ciliary oscillations near the instability threshold

Debasmita Mondal, Ronojoy Adhikari, Prerna Sharma*

*Corresponding author. Email: prerna@iisc.ac.in

Published 12 August 2020, *Sci. Adv.* **6**, eabb0503 (2020)
DOI: 10.1126/sciadv.abb0503

The PDF file includes:

Sections S1 to S5
Legend for movie S1
Table S1
Figs. S1 to S4
References

Other Supplementary Material for this manuscript includes the following:

(available at advances.sciencemag.org/cgi/content/full/6/33/eabb0503/DC1)

Movie S1

Supplementary Materials: Internal friction controls active ciliary oscillations near the instability threshold

Section S1. Tension forces in the filament

The tangential component of the stress resultant is the tension within the filament (*40*), which we can compute from filament velocity as $F_t(s) = \mathbf{t}(s) \cdot \int_s^L \mathbf{f}^v(s')ds' = -\mathbf{t}(s) \cdot \int_s^L \gamma \cdot \dot{\mathbf{R}}(s')ds' = -\gamma_n g_t(s)$, where, $g_t(s) = \mathbf{t}(s) \cdot \int_s^L [\dot{R}_n(s')\mathbf{n}(s') + (\dot{R}_t(s')/2)\mathbf{t}(s')]ds'$. The tension force in the filament is also too small (fig. S3) compared to the internal elastic forces.

Section S2. Linear stability analysis

We Fourier transform the coupled equations of motion [Eq. 7 in main text] in space and time with the following convention

$$\Delta\theta(s,t) = \int \frac{dz}{2\pi} \sum_n \widetilde{\Delta\theta}(q_n, z) e^{i(q_n s - zt)}, \quad \text{[similarly for } m^{\mathcal{A}}(s,t) \to \widetilde{m^{\mathcal{A}}}(q_n, z)\text{]}$$

where q_n is the discretized wavenumber of the nth mode due to finite length of the filament and z is the complex frequency. In matrix form,

$$\begin{bmatrix} (1+q_n^2) - iz(q_n^2 + \nu_\kappa/\nu_u) & -1 \\ b_3 & iz - b_1 \end{bmatrix} \begin{bmatrix} \widetilde{\Delta\theta} \\ \widetilde{m^{\mathcal{A}}} \end{bmatrix} = \begin{bmatrix} 0 \\ 0 \end{bmatrix} \tag{S1}$$

For non-trivial solution, the determinant of the above matrix must be zero. Hence the dispersion relation is

$$\left[(1+q_n^2) - iz(q_n^2 + \nu_\kappa/\nu_u)\right](iz - b_1) + b_3 = 0 \tag{S2}$$

This is a quadratic equation in the complex frequency z of the form $A(q_n)z^2 + B(q_n)z + C(q_n) = 0$ whose coefficients are $A(q_n) = q_n^2 + \nu_\kappa/\nu_u$, $B(q_n) = i\left[(1+q_n^2) + b_1(q_n^2 + \nu_\kappa/\nu_u)\right]$ and $C(q_n) = b_3 - b_1(1+q_n^2)$. Roots are $z_{1,2} = \left[-B(q_n) \pm \sqrt{B^2(q_n) - 4A(q_n)C(q_n)}\right]/2A(q_n)$. As the time dependent component in the solution is e^{-izt}, existence of the real part of the root

will imply that the solution is oscillatory and sign of the imaginary part of z will decide if the solution is growing or decaying. Hence, the conditions for unstable oscillations are $Im[z] > 0$ and $Re[z] \neq 0$. The frequency of the oscillations is therefore given by $\omega = -Re[z]$.

Section S3. Why strain softening and shear thinning facilitates the instability to oscillations?

We Fourier transform the dynamical equation of motion (Eq. 6 in the main text) in space and time under oscillating shear of fundamental frequency ω i.e. $\Delta\theta \sim \widetilde{\Delta\theta} e^{i(q_n s+\omega t)}$ where $\omega = -Re(z)$ is real and q_n is the discretized wavenumber of the nth mode.

$$(-q_n^2 - i\omega q_n^2 - 1 - i\omega \nu_\kappa/\nu_u)\widetilde{\Delta\theta} + \widetilde{m^{\mathcal{A}}} = 0 \tag{S3}$$

Replacing the fundamental Fourier mode of active stress as $\widetilde{m^{\mathcal{A}}} = (G' + i\omega G'')\widetilde{\Delta\theta}$, where where G' and G'' corresponds to elastic and viscous response of the system, respectively and are related to b_1, b_3 such that $b_1, b_3 < 0 \implies G', G'' > 0$ (see main text), we obtain

$$\underbrace{-(1+q_n^2)\widetilde{\Delta\theta}}_{\text{passive elastic}} \underbrace{-i\omega(q_n^2 + \nu_\kappa/\nu_u)\widetilde{\Delta\theta}}_{\text{passive viscous}} + \underbrace{(G' + i\omega G'')\widetilde{\Delta\theta}}_{\text{active elastic + viscous}} = 0 \tag{S4}$$

Hence, the passive elastic and viscous terms in the equation of motion are negative i.e. they resist the sliding caused by the active dynein motors. Now, if $G', G'' < 0$, the system's passive spring constant and friction coefficient get renormalized by the ATP dependent dynein activity and thus, both the active and passive components of the system resist the sliding ultimately leading to a quiescent stable state of the filament. On the other hand, if $G', G'' > 0$, dynein motors work against the material response so that the system becomes unstable and undergoes oscillations. Here, $G' > 0$ indicates that elastic stresses reduce within the axoneme as motor activity increases which is called 'strain softening' and $G'' > 0$ indicates that the axoneme becomes less viscous with increasing motor activity which is called 'shear thinning'.

We note that nonlinear viscoelastic effects are not needed for an active material, such as the axoneme, to shear thin/strain soft, because the nonequilibrium active stresses generated by the dynein motors can produce structural rearrangements within the axoneme by binding and unbinding the dynein cross-bridges across microtubule doublets. This is in contrast to passive equilibrium systems which have to be intrinsically nonlinear to strain soft/shear thin under large external shear as thermal energy alone is insufficient to drive structural rearrangements in such systems.

Section S4. Comparison with microscopic load dependent detachment model of dynein motors

In the microscopic load dependent detachment model, the motor detachment rate (k_{off}) is assumed to increase exponentially with increasing load (i.e. single motor force, f_+), which in turn decreases linearly with the dynein sliding speed (v_d) by the force-velocity relationship of the motors (*10,33,41*).

$$k_{off}(f_+) = k_0 \exp\left[\frac{f_+}{f_c}\right] = k_0 \exp\left[\frac{\bar{f} - f' v_d}{f_c}\right] \tag{S5}$$

where $\bar{f}$ is the dynein stall force, f' is the slope of the dynein force-velocity curve, f_c is the characteristic unbinding force generally given by $f_c \approx \bar{f}/2$ (*10,33*). The force-velocity slope is given by $f' = \bar{f}/v_0$, where v_0 is the dynein velocity at zero load. In the limit of low sliding speed i.e. $\frac{f' v_d}{f_c} << 1$, the above exponential relation linearizes to

$$k_{off}(f_+) = \bar{k}_{off}\left[1 - \frac{f' v_d}{f_c}\right] \tag{S6}$$

where $\bar{k}_{off} = k_{off}(\bar{f}) = k_0 \exp(\bar{f}/f_c)$ is the motor detachment rate at stall [refer to Eq. B4 in Appendix B of (*10*)]. Now let us see if our experiments near the critical ATP concentration satisfy the linearizing condition, $\frac{f' v_d}{f_c} << 1 \implies \frac{v_d}{v_0/2} << 1$.

Axoneme being a cross-linked filament, the angular speed of the axoneme ($\partial_t \Delta\theta$) is related

to the sliding speed per dynein motor as $v_d = a_{MT}\partial_t\Delta\theta/L\rho\bar{p}$ (*10*). Here $a_{MT} = 24$ nm is the MT interdoublet spacing in which the dyneins work (*4*), L is the filament length at which angular speed is calculated from experiments, $\rho = 198$ μm^{-1} is motor density (*4*) and $\bar{p} = 0.02$ is the fraction of motor domains that are attached to MT, equivalent to the duty ratio (*21,33*). Therefore, $L\rho\bar{p}$ is the total number of motors bound to a single MT along the length of the filament. At 60 μM ATP, $\partial_t\Delta\theta \approx 80$ rad/s (from Fig. 4E of main text) at $L \approx 9$ μm. Using these values $v_d \approx 54$ nm/s. Earlier experiments have measured the zero load dynein velocity at 60 μM ATP to be $v_0 \approx 2$ μm/s (*42,21*). Hence the ratio of dynein sliding speed in our experiments to the half of its zero load velocity $\dfrac{v_d}{v_0/2} \approx 0.05 << 1$. To summarize, the axoneme beating near the instability threshold at 60 μM ATP is in the dominantly linear regime (equivalently weakly nonlinear regime) of the post-bifurcation dynamics. Therefore, our choice of linear constitutive relationship for the active moment and the associated linear stability analysis is valid near the instability threshold [also refer to Fig. 3a and associated text of (*33*)].

Now, that we have shown our experiments are consistent with the condition for linearizing the exponential dependence of motor detachment rate on sliding speed, we connect our constitutive equation for active moment to this linearized version of microscopic motor dynamics model (*sliding control motor coordination model*) proposed by Riedel-Kruse et al. (*10*). In (*10*), the active shear force per unit length is related to the shear displacement by a response function, $\chi = K + i\omega\lambda$, as per their notation. The sign convention of G' and G'' is opposite to the elasto-viscous response coefficients (K and λ) of (*10*) [also of (*19,32*)], as active drive in these references has opposite sign convention. The equivalence of our response coefficients to their microscopic model of load dependent detachment of motors [refer to Eq. B9 in Appendix B of (*10*)] are as follows:

(a) $G' \equiv -a^2K = 2a^2\rho\bar{f}\frac{f'}{f_c}\bar{p}(1-\bar{p})\frac{\omega^2\tau}{1+(\omega\tau)^2}$. This quantity is always positive.

(b) $G'' \equiv -a^2\lambda = -2a^2\rho f'\bar{p}\big[1-\frac{\bar{f}}{f_c}\frac{(1-\bar{p})}{\{1+(\omega\tau)^2\}}\big]$. This quantity can be positive or negative.

In the above expressions, $\bar{\tau}$ is the relaxation time of motor attachment/detachment and all other variables are already defined in the preceding paragraph. The sign in (b) depends on $\bar{\tau}$ and $\bar{p}$ as: (i) $G'' > 0$ for $\omega\bar{\tau} << 1$ and $\bar{p} \sim 0$ and (ii) $G'' < 0$ for $\omega\bar{\tau} >> 1$ and $\bar{p} \sim 1$. For our active filament $G'' > 0$ i.e. $b_3 < 0$ asserts that $\omega\bar{\tau} << 1$ and $\bar{p} \sim 0$. This means that the axonemal dynein motors are short lived with low duty ratio, which agrees with (*21*).

Section S5. Estimates of Γ_u from literature

All parameters, except the shear friction coefficient, have been experimentally measured for an axoneme or at least for microtubules in the existing literature as mentioned in the main text. There is uncertainty in the value of Γ_u. Following (table S1) are the values used for this coefficient for constructing active filament models in the literature, except for the last entry which is an experimental study on shear elasticity.

We note that the exact value of this coefficient does not affect the existence of oscillation in our theoretical model (fig. S4) rather it modulates the magnitudes of the viscoelastic response coefficients of the active stress. The sliding friction coefficient Γ_u appears in the dimensionless dynamical equation as the ratio ν_κ/ν_u. Variation of ν_κ/ν_u implies variation in Γ_u because all parameters except Γ_u are experimentally known for an axoneme/MT. Figure S4 shows that unstable oscillations exists for $\nu_\kappa/\nu_u \in [0.1,\ 50]$. Correspondingly, the varation of Γ_u in this range is $[10^{-7},\ 0.5 \times 10^{-4}]$ Ns/m.

Movie caption

Movie S1. Movie of an isolated and reactivated axoneme in presence of tracers. High speed phase contrast movie of a reactivated and clamped *Chlamydomonas* axoneme at 60 μM ATP in presence of 200 nm tracer particles. Scale bar, 5 μm.

Reference	Γ_u [$\times 10^{-6}$] Ns/m	Comparison of shear friction with elasticity
Brokaw (*28*)	0.06-0.51	negligible compared to elastic terms
Murase-Shimizu (*29*)	30	very high compared to elastic terms, overdamping the system
Bayly-Wilson (*43*)	0.5	negligible compared to elastic terms
Bayly-Dutcher (*31*)	0.16	negligible compared to elastic terms
**Ingmar Riedel** (*27*)	**10**	**comparable and competing with elastic terms,** ✓
Minoura-Yagi-Kamiya (*24*)	365	very high compared to elastic terms, overdamping the system*

*Obtained from digitizing Fig. 5 of (*24*), and fitting a creep response function for Kelvin-Voigt model of viscoelasticity.

Table S1: **Possible values of shear friction coefficient from the literature.** Shear friction coefficient from existing literature and reasons for neglecting/accepting them.

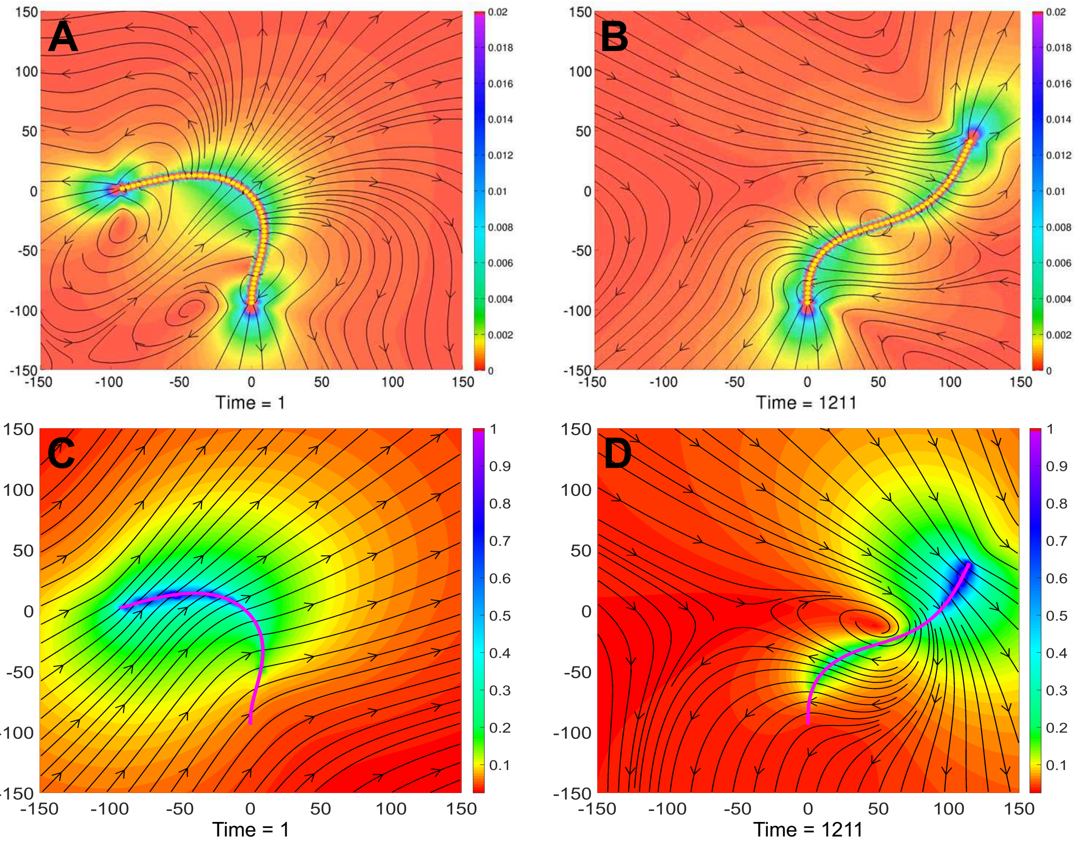

Fig. S1: **Active filaments driven by slip at boundary vs driven internally by motors.** (**A**, **B**) Flow fields of a planar flexible beating of a clamped filament, consisting of chemomechanically active beads, at two instants of the oscillation cycle, adapted from Supplementary video 2 in (*36*). (**C**, **D**) Computed flow fields using slender body approximation and resistive force theory (unbounded flow) where the filament positions were extracted from the video. The mismatch between (A-C) and (B-D) imply that the filament must not be internally driven instead slip driven as expected of a phoretic chain. The colorbars to the right of (C) and (D) represents the normalized speed.

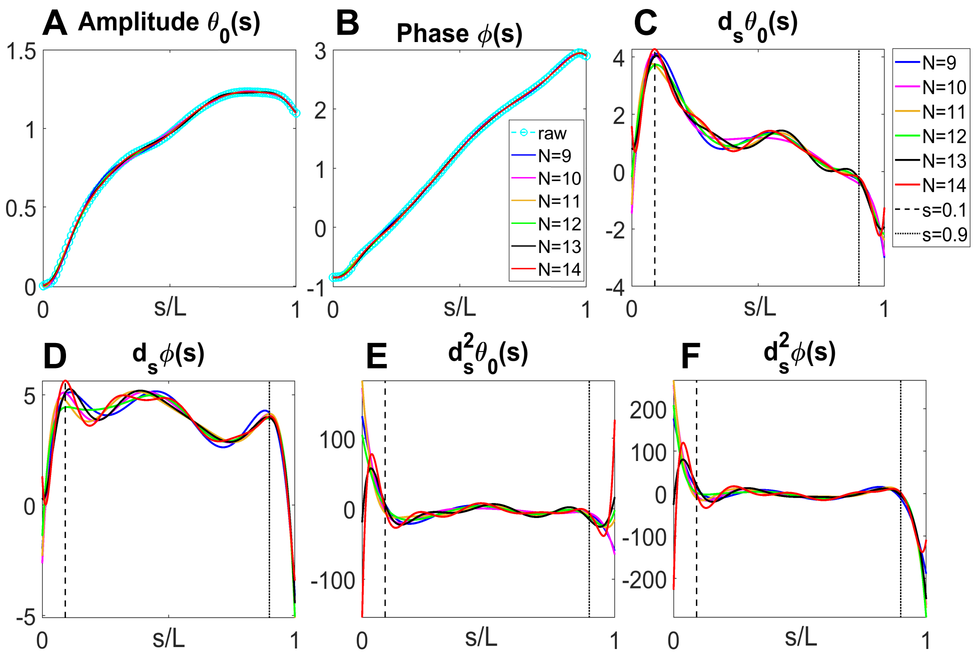

Fig. S2: **Chebyshev differentiation of traveling wave parameters.** (**A**) Amplitude (θ_0) and (**B**) phase (ϕ) of the traveling wave parameterization to θ are plotted in cyan circles. Interpolation to them for different Chebyshev polynomial orders N almost converges to the actual value. First order Chebyshev differentiation of (**C**) θ_0 and (**D**) ϕ with respect to s for different polynomial orders. Second order Chebyshev differentiation of (**E**) θ_0 and (**F**) ϕ with respect to s for different polynomial orders. Legends of (C-F) are shown beside (C).

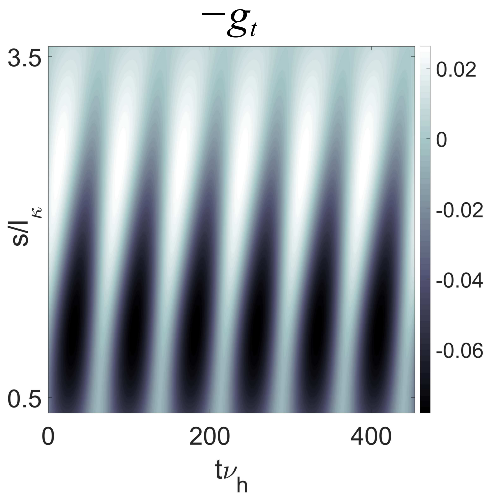

Fig. S3: **Tension forces in the filament.** Space-time plot of the tension force i.e. tangential component of the stress resultant, along the filament for three beat cycles. The length and time scales are l_κ and $1/\nu_h$. The colorbar represents its magnitude, with the force scale $EI/l_\kappa^2 = 80$ pN.

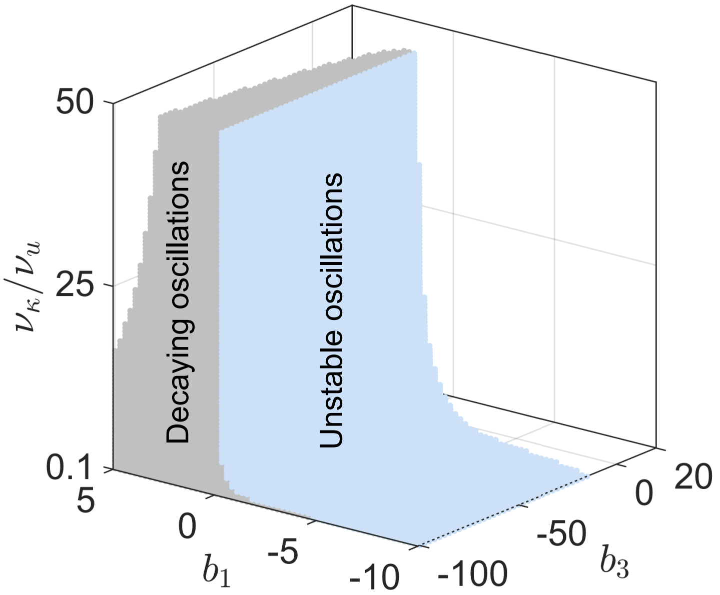

Fig. S4: **Existence of oscillations with varying sliding friction coefficient.** Oscillatory nature of the complex frequency, z in the parameter space $(b_3, b_1, \nu_\kappa/\nu_u)$ for the fundamental mode, q_1. No oscillatory solutions exist in the white regions. The range of $\nu_\kappa/\nu_u \in [0.1,\ 50]$ corresponds to $\Gamma_u \in [10^{-7},\ 0.5 \times 10^{-4}]$ Ns/m (considering the value of ν_κ to be fixed at 375 Hz). The blue region of unstable oscillations for all these values of Γ_u indicate that the magnitude of Γ_u does not affect the existence of oscillations.

REFERENCES AND NOTES

1. R. E. Goldstein, Green algae as model organisms for biological fluid dynamics. *Annu. Rev. Fluid Mech.* **47**, 343–375 (2015).

2. E. A. Gaffney, H. Gadêlha, D. J. Smith, J. Blake, J. Kirkman-Brown, Mammalian sperm motility: Observation and theory. *Annu. Rev. Fluid Mech.* **43**, 501–528 (2011).

3. M. C. Marchetti, J. F. Joanny, S. Ramaswamy, T. B. Liverpool, J. Prost, M. Rao, R. A. Simha, Hydrodynamics of soft active matter. *Rev. Mod. Phys.* **85**, 1143–1189 (2013).

4. D. Nicastro, C. Schwartz, J. Pierson, R. Gaudette, M. E. Porter, J. R. McIntosh, The molecular architecture of axonemes revealed by cryoelectron tomography. *Science* **313**, 944–948 (2006).

5. R. Dreyfus, J. Baudry, M. L. Roper, M. Fermigier, H. A. Stone, J. Bibette, Microscopic artificial swimmers. *Nature* **437**, 862–865 (2005).

6. W. F. Paxton, K. C. Kistler, C. C. Olmeda, A. Sen, S. K. St. Angelo, Y. Cao, T. E. Mallouk, P. E. Lammert, V. H. Crespi, Catalytic nanomotors: Autonomous movement of striped nanorods. *J. Am. Chem. Soc.* **126**, 13424–13431 (2004).

7. C. J. Brokaw, Bend propagation by a sliding filament model for flagella. *J. Exp. Biol.* **55**, 289–304 (1971).

8. R. Rikmenspoel, The equation of motion for sperm flagella. *Biophys. J.* **23**, 177–206 (1978).

9. J. J. Blum, M. Hines, Biophysics of flagellar motility. *Q. Rev. Biophys.* **12**, 103–180 (1979).

10. I. H. Riedel-Kruse, A. Hilfinger, J. Howard, F. Jülicher, How molecular motors shape the flagellar beat. *HFSP J.* **1**, 192–208 (2007).

11. P. Bayly, K. Wilson, Analysis of unstable modes distinguishes mathematical models of flagellar motion. *J. R. Soc. Interface* **12**, 20150124 (2015).

12. P. Sartori, V. F. Geyer, A. Scholich, F. Jülicher, J. Howard, Dynamic curvature regulation accounts for the symmetric and asymmetric beats of chlamydomonas flagella. *eLife* **5**, e13258 (2016).

13. R. E. Goldstein, M. Polin, I. Tuval, Noise and synchronization in pairs of beating eukaryotic flagella. *Phys. Rev. Lett.* **103**, 168103 (2009).

14. V. F. Geyer, F. Jülicher, J. Howard, B. M. Friedrich, Cell-body rocking is a dominant mechanism for flagellar synchronization in a swimming alga. *Proc. Natl. Acad. Sci. U.S.A.* **110**, 18058–18063 (2013).

15. G. Quaranta, M.-E. Aubin-Tam, D. Tam, Hydrodynamics versus intracellular coupling in the synchronization of eukaryotic flagella. *Phys. Rev. Lett.* **115**, 238101 (2015).

16. K. Y. Wan, R. E. Goldstein, Coordinated beating of algal flagella is mediated by basal coupling. *Proc. Natl. Acad. Sci. U.S.A.* **113**, E2784–E2793 (2016).

17. J. Elgeti, G. Gompper, Emergence of metachronal waves in cilia arrays. *Proc. Natl. Acad. Sci. U.S.A.*

110, 4470–4475 (2013).

18. M. G. Poirier, J. F. Marko, Effect of internal friction on biofilament dynamics. *Phys. Rev. Lett.* **88**, 228103 (2002).

19. S. Camalet, F. Jülicher, Generic aspects of axonemal beating. *New J. Phys.* **2**, 24.1–24.23 (2000).

20. J. Ericksen, C. Truesdell, Exact theory of stress and strain in rods and shells. *Arch. Ration. Mech. Anal.* **1**, 295–323 (1957).

21. J. Howard, *Mechanics of Motor Proteins and the Cytoskeleton* (Sinauer Associates, Publishers, 2001).

22. J. R. Blake, A note on the image system for a stokeslet in a no-slip boundary. *Math. Proc. Camb. Philos. Soc.* **70**, 303–310 (1971).

23. G. Xu, K. S. Wilson, R. J. Okamoto, J.-Y. Shao, S. K. Dutcher, P. V. Bayly, Flexural rigidity and shear stiffness of flagella estimated from induced bends and counterbends. *Biophys. J.* **110**, 2759–2768 (2016).

24. I. Minoura, T. Yagi, R. Kamiya, Direct measurement of inter-doublet elasticity in flagellar axonemes. *Cell Struct. Funct.* **24**, 27–33 (2001).

25. C. P. Brangwynne, G. H. Koenderink, E. Barry, Z. Dogic, F. C. MacKintosh, D. A. Weitz, Bending dynamics of fluctuating biopolymers probed by automated high-resolution filament tracking. *Biophys. J.* **93**, 346–359 (2007).

26. M. E. Janson, M. Dogterom, A bending mode analysis for growing microtubules: Evidence for a velocity-dependent rigidity. *Biophys. J.* **87**, 2723–2736 (2004).

27. I. Riedel, Mechanics of the axoneme: Self-organized beating patterns and vortex arrays of spermatozoa, Ph.D. thesis, Ph. D. dissertation, TU Dresden (2005).

28. C. J. Brokaw, Computer simulation of flagellar movement: I. demonstration of stable bend propagation and bend initiation by the sliding filament model. *Biophys. J.* **12**, 564–586 (1972).

29. M. Murase, H. Shimizu, A model of flagellar movement based on cooperative dynamics of dynein-tubulin cross-bridges. *J. Theor. Biol.* **119**, 409–433 (1986).

30. M. Hines, J. Blum, Bend propagation in flagella. i. derivation of equations of motion and their simulation. *Biophys. J.* **23**, 41–57 (1978).

31. P. V. Bayly, S. K. Dutcher, Steady dynein forces induce flutter instability and propagating waves in mathematical models of flagella. *J. R. Soc. Interface* **13**, 20160523 (2016).

32. S. Camalet, F. Jülicher, J. Prost, Self-organized beating and swimming of internally driven filaments. *Phys. Rev. Lett.* **82**, 1590–1593 (1999).

33. D. Oriola, H. Gadêlha, J. Casademunt, Nonlinear amplitude dynamics in flagellar beating. *R. Soc. Open Sci.* **4**, 160698 (2017).

34. B. Qin, A. Gopinath, J. Yang, J. P. Gollub, P. E. Arratia, Flagellar kinematics and swimming of algal cells in viscoelastic fluids. *Sci. Rep.* **5**, 9190 (2015).

35. C. J. Brokaw, Effects of viscosity and atp concentration on the movement of reactivated sea-urchin sperm flagella. *J. Exp. Biol.* **62**, 701–719 (1975).

36. A. Laskar, R. Singh, S. Ghose, G. Jayaraman, P. S. Kumar, R. Adhikari, Hydrodynamic instabilities provide a generic route to spontaneous biomimetic oscillations in chemomechanically active filaments. *Sci. Rep.* **3**, 1964 (2013).

37. J. Alper, V. Geyer, V. Mukundan, J. Howard, Reconstitution of flagellar sliding. *Methods Enzymol.* **524**, 343–369 (2013).

38. K.-i. Wakabayashi, R. Kamiya, Axonemal motility in Chlamydomonas. *Methods Cell Biol.* **127**, 387–402 (2015).

39. D. Favre, B. Muellhaupt, Reactivation of creatine kinase by dithiothreitol prior to use in an in vitro translation extract. *ALTEX* **22**, 259–264 (2005).

40. S. G. Prasath, J. Marthelot, R. Govindarajan, N. Menon, Relaxation of a highly deformed elastic filament at a fluid interface. *Phys. Rev. Fluids* **1**, 033903 (2016).

41. B. Chakrabarti, D. Saintillan, Hydrodynamic synchronization of spontaneously beating filaments. *Phys. Rev. Lett.* **123**, 208101 (2019).

42. E. Yokota, I. Mabuchi, C/a dynein isolated from sea urchin sperm flagellar axonemes. enzymatic properties and interaction with microtubules. *J. Cell Sci.* **107**, 353–361 (1994).

43. P. V. Bayly, K. S. Wilson, Equations of interdoublet separation during flagella motion reveal mechanisms of wave propagation and instability. *Biophys. J.* **107**, 1756–1772 (2014).
